# Synthesis and pharmacological evaluation of pyrazolopyrimidopyrimidine derivatives: anti-inflammatory agents with gastroprotective effect in rats

**DOI:** 10.1007/s00044-013-0742-x

**Published:** 2013-09-04

**Authors:** Amine Karoui, Fatma Allouche, Monia Deghrigue, Asma Agrebi, Abderrahman Bouraoui, Fakher Chabchoub

**Affiliations:** 1 Laboratoire de Chimie Appliquée, Hétérocycles, Corps Gras et Polymères, Faculté des Sciences de Sfax, Université de Sfax, 3018 Sfax, Tunisia; 2 Unité de Recherche des Substances Actives Marines (URSAM), Laboratoire de Pharmacologie, Faculté de Pharmacie, Université de Monastir, 5000 Monastir, Tunisia

**Keywords:** Aminocyanopyrazole, Anti-inflammatory, Gastroprotective, Pyrazolo[3,4-*d*]pyrimidine, Dihydropyrazolo[3′,4′:4,5]pyrimido[1,6-*a*]pyrimidine

## Abstract

We report the synthesis of new anti-inflammatory 1,7-dihydropyrazolo[3′,4′:4,5]pyrimido[1,6-*a*]pyrimidine **5** from aminocyanopyrazole. All compounds were characterized by physical, chemical and spectral studies. Preliminary pharmacological evaluation of the resulting products showed that compounds **5a**, **b**, **f** (50–100 mg/kg, i.p) are active anti-inflammatory agents in carrageenan-induced rat paw oedema assay, and their effects are comparable to that of acetylsalicylic–lysine (300 mg/kg, i.p.), used as a reference drug. The nature of substituent (Y, R_3_) had a pronounced effect on the anti-inflammatory activity. Studies of structure–activity relationships have led to selection of compound ethyl-3,5-dimethyl-7-imino-*N* ^1^-phenyl-1,7-dihydropyrazolo[3′,4′:4,5]pyrimido[1,6-*a*]pyrimidine-6-carboxylate, **5f** which exhibited the most potent anti-inflammatory activity. In addition, the compounds **5a**, **b**, **f** showed a significant gastroprotective effect against HCl/EtOH-induced gastric ulcer.

## Introduction

Nonsteroidal anti-inflammatory drugs (NSAIDs) are most widely used to treat variety of acute and chronic inflammatory diseases. Such drugs are being increasingly used for the treatment of postoperative pain (Moote, 1992) with or without supplemental opioid agents. The pharmacological action of these agents was assigned to inhibit two enzymes, known as cyclooxygenase-1 (COX-1) and cyclooxygenase-2 (COX-2) (Vane *et al.*, 1998). The constitutive isoform COX-1 is present in most tissues and is involved in the synthesis of prostaglandins vital to normal cell function. In contrast, the inducible isoform COX-2 appears to be produced primarily in response to growth factors or inflammatory mediators, such as cytokines (Vane and Botting, 1996). Many of the currently available NSAIDs, including indomethacin and piroxicam, are more potent inhibitors of COX-1 than that of COX-2 (Vane and Botting, 1995). This preferential inhibition of COX-1 may be responsible for many of the adverse effects associated with NSAIDs. It has been postulated that NSAIDs which preferentially inhibit COX-2, such as meloxicam (Lipscomb *et al.*, 1998), celecoxib (Simon *et al.*, 1998) and several experimental drugs including NS 398, L-745,337 and DFP, should produce the same or better anti inflammatory effects with less gastrointestinal, haematological and renal toxicities than classical NSAIDs (Winter *et al.*, 1962). Pyrazolopyrimidines are a class of sedative and anxiolytic drugs such as Zaleplon known by its hypnotic effect (Weitzel *et al.*, 2000). However, pyrazolopyrimidine derivatives become a new chemical resource for searching of novel bioactive compounds in drug development.

On this basis, we directed our attention to the synthesis of novel 1,7-dihydropyrazolo[3′,4′:4,5]pyrimido[1,6-*a*]pyrimidines **5a**–**i** related to aminocyanopyrazole with the aim of improving their anti-inflammatory activity and reducing their ulcerogenic properties as it appeared to be plausible that variation of the active compound structures could exert a pronounced influence on activity, as the case with **5b**, **f**.

## Materials and methods

### Chemistry

Phenyl hydrazine, malononitrile, triethylorthoester and ammoniac were purchased from Sigma Chemical (Berlin, Germany). Analytical grade solvents (ethanol, HCl, ethyl acetate, chloroform) were obtained from Merck.

Melting points (mp) were determined on a Buchi capillary apparatus and were uncorrected. Nuclear magnetic resonance (NMR) spectra were recorded on a Bruker 300 spectrometer (^1^H at 300 MHz and ^13^C at 75 MHz) with deuterio-dimethylsulphoxide (*d*-DMSO) as solvent and tetramethylsilane as internal standard reference. Infra-red (IR) spectra were recorded on a Bio-rad FTS-6000 spectrometer. Solvents used in reactions were dried and distilled before use. The purity of all synthesized compounds was controlled by thin layer chromatography (TLC; Merck silica gel plates 60F-254). High resolution masses were recorded on a spectrometer JEOL JMS-Gcmate II is composed of a GC/MS system from compounds dissolved in dichloromethane.

#### Synthesis and spectral data of compounds **2–5**

##### 5-Amino-4-cyano-*N*^1^-phenyl pyrazoles (**2**)

5-Amino-4-cyano-1-*N* ^1^-phenyl pyrazoles prepared via a standard addition of hydrazine derivatives to ketene ethoxymethylene compounds following the reported procedure. Recrystallization from ethanol afforded pure **2** in good yields.

##### 4-Cyano-*N*^1^-phenyl pyrazolo-5-imidates (**3**)

The required 5-amino-4-cyano-*N* ^*1*^-phenyl pyrazole (1.0 mmol) was treated with triethylorthoester 6.0 mmol) and a catalytic amount of acetic acid and the mixture was refluxed for 24 h. After cooling, the reaction mixture was evaporated. The product was filtered, washed with diethyl ether then purified by recrystallisation (ethanol) (Gupta *et al.*, 2008; Allouche *et al.*, 2013).

##### 4-Amino-*N*^1^-phenyl pyrazolo[3,4-*d*]pyrimidine (**4**)

A solution of imidate **3** (1.0 mmol) in dry ethanol (5 ml) was treated with ammoniac (2.0 mmol) and a catalytic amount of acetic acid. The reaction mixture was refluxed for 6 h, and the formed solid was collected by filtration, dried and recrystallized from ethanol to give compound **4**.

- *4-Amino-N* ^*1*^ *-phenyl-1H-pyrazolo[3,4-d]pyrimidine* ***4a*** Yield 83 %; mp 228 °C; IR (cm^−1^); *ν*_NH2_ 3100, 3283; *ν*_C=N_ 1480, 1500, 1590 cm^−1^; RMN ^1^H (*δ* ppm, DMSO): 4.69 (2H, s, NH_2_), 7.36 (1H, t, *J* = 7.3 Hz, ArH_4_), 7.48 (2H, t, *J* = 7.3 Hz, ArH_3_ and ArH_5_), 7.52 (2H, d, *J* = 7.3 Hz, ArH_2_ and ArH_6_), 7.60 (1H, s, H_3_), 7.72 (1H, s, H_6_), ^13^C RMN (*δ* ppm, DMSO): 114.14 (C-3a), 124.27 (C-2′ and C-6′), 129.00 (C-4′), 129.58 (C-3′ and C-5′), 130.04 (C-3), 136.94 (C-1′), 141.36 (C-7a), 149.83 (C-6), 156.84 (C-4); HRMS Calcd. for C_11_H_9_N_5_: 211.0858, found: 211.0859.
- *4-Amino-3-methyl-N* ^*1*^ *-phenyl-1H-pyrazolo[3,4-d]pyrimidine* ***4b*** Yield 68 %; mp 192 °C; IR (cm^−1^); *ν*_NH2_ 3083, 3317; *ν*_C=N_ 1626, 1647, 1665; RMN ^1^H (*δ* ppm, DMSO): 2.76 (3H, s, CH_3_), 5.97 (2H, s, NH_2_), 7.33 (1H, t, *J* = 7.1 Hz, ArH_4_), 7.57 (2H, t, *J* = *7*.1 Hz, ArH_3_ and ArH_5_), 8.16 (2H, d, *J* = *7*.1 Hz, ArH_2_ and ArH_6_), 8.46 (1H, s, H_3_); RMN^13^C (*δ* ppm, DMSO):14.89 (CH_3_),101.23 (C-3a), 121.49 (C-2′ and C-6′), 126.37 (C-4′), 129.19 (C-3′ and C-5′), 138.81 (C-3), 141.83 (C-1′), 154.41 (C-7a), 156.48 (C-4), 158.40 (C-6); HRMS Calcd. for C_12_H_11_N_5_: 225.1014, found: 225.1018.
- *4-Amino-6-methyl-N* ^*1*^ *-phenyl-1H-pyrazolo[3,4-d]pyrimidine* ***4c*** Yield 70 %; mp 160 °C; IR (cm^−1^); *ν*_NH2_ 3090, 3320; *ν*_C=N_ 1597, 1638, 1663; RMN ^1^H (*δ* ppm, DMSO): 2.65 (3H, s, CH_3_), 4.28 (2H, s, NH_2_), 7.28 (1H, t, *J* = 7.3 Hz, ArH_4_), 7.56 (2H, t, *J* = 7.3 Hz, ArH_3_ and ArH_5_), 8.19 (2H, d, *J* = 7.3 Hz, ArH_2_ and ArH_6_), 8.29 (1H, s, H_6_); RMN^13^C (*δ* ppm, DMSO): 14.44 (CH_3_), 100.24 (C-3a), C_arom_ 120.24 (C-2′ and C-6′), 124.67 (C-4′), 129.16 (C-3′ and C-5′), 138.8 (C-3), 142.79 (C-1′); C_3 _154.14 (C-7a), 156.51 (C-4),158.58 (C-6); HRMS Calcd. for C_12_H_11_N_5_ : 225.1014, found: 225.1016.

##### 7-Imino-*N*^1^-phenyl-1,7-dihydropyrazolo[3′,4′:4,5]pyrimido[1,6-*a*]pyrimidine **5a**–**e**

A mixture of compound **4** (1.0 mmol), ketene ethoxymethylene compounds **1** or ethyl-2-cyano-3-ethoxyalkyl-2-enoate (1.0 mmol) and a catalytic amount of acetic acid was refluxed for 2 h in 10 ml ethanol. The formed precipitate was filtered, washed by diethyl ether, dried and recrystallized from ethanol to give compound **5** in good yield.

- *6-Cyano-7-imino-3-methyl-N* ^*1*^ *-phenyl-1,7-dihydropyrazolo[3′,4′:4,5]pyrimido[1,6-a]pyrimidine* ***5a*** Yield 68 %; mp 290 °C; IR (cm^−1^); *ν*_NH_ 3356; *ν*_C≡N_ 2212; *ν*_C=N_ 1534, 1554, 1587; RMN ^1^H (*δ* ppm, DMSO): 2.51 (3H, s, CH_3_); 7.38 (1H, t, *J* = 7.3 Hz, ArH_4_); 7.53 (2H, t, *J* = 7.3 Hz, ArH_3_ and ArH_5_); 7.71 (2H, d, *J* = 7.3 Hz, ArH_2_ and ArH_6_); 8.02 (1H, s, H_5_); 8.38 (1H, s, H_9_); 8.66 (1H, s, NH); RMN^13^C (*δ* ppm, DMSO): 14.64 (CH_3_); 91.81 (C-6); 105.88 (C-3a); 116.24 (CN); C_arom_ 120.46 (C-2′ and C-6′), 124.17 (C-4′), 129.27 (C-3′ and C-5′), 137.89 (C-1′),143.42 (C-10a), 149.71 (C-3),159.61 (C-5),161.88 (C-9), 162.15 (C-4a); 163.43 (C-7); HRMS Calcd. for C_16_H_11_N_7_ :301.1076, found: 301.1051.
- *6-Cyano-7-imino-3,5-dimethyl-N* ^*1*^ *-phenyl-1, 7-dihydropyrazolo[3′, 4′:4, 5]pyrimido[1, 6-a]pyrimidine* ***5b*** Yield 54 %; mp 182 °C; IR (cm^−1^): *ν*_NH_ 3324; *ν*_C≡N_ 2230; *ν*_C=N_ 1509, 1562, 1586; RMN ^1^H (*δ* ppm, DMSO): 2.50 (3H, s, CH_3_), 2.64 (3H, s, CH_3_); 7.26 (1H, t, *J* = 7.3 Hz, ArH_4_); 7.51 (2H, t, *J* = 7.3 Hz, ArH_3_ and ArH_5_); 7.54 (2H, d, *J* = 7.3 Hz, ArH_2_ and ArH_6_); 8.19 (1H, s, H_9_); 8.27 (1H, s, NH); RMN^13^C (*δ* ppm, DMSO): 14.42 (CH_3_); 21.00 (CH_3_)_;_ 87.23 (C-6); 100.25 (C-3a); 109.00 (CN); 120.22 (C-2′ and C-6′), 125.51 (C-4′), 128.98 (C-3′ and C-5′), 138.89 (C-1′); 142.79 (C-10a); 154.17 (C-3), 156.49 (C-5), 164.59 (C-9), 165.71 (C-4a), 167.94 (C-7); HRMS Calcd. for C_17_H_13_N_7_ : 315.1232, found: 315.1214.
- *6-Cyano-7-imino-9-methyl-N* ^*1*^ *-phenyl-1,7-dihydropyrazolo[3′,4′:4,5]pyrimido[1,6-a]pyrimidine* ***5c*** Yield 71 %; mp 166 °C; IR (cm^−1^); *ν*_NH_ 3321.86; *ν*_C≡N_ 2223, 1536, 1561, 1599; RMN ^1^H (*δ* ppm, DMSO): 2.62 (3H, s, CH_3_); 7.40 (1H, t, *J* = 7.3 Hz, ArH_4_); 7.49 (2H, t, *J* = 7.3 Hz, ArH_3_ and ArH_5_); 7.68 (2H, d, *J* = 7.3 Hz, ArH_2_ and ArH_6_); 8.19 (1H, s, H_5_); 8.41 (1H, s, H_9_); 8.73 (1H, s, NH); RMN^13^C (*δ* ppm, DMSO): 14.32 (CH_3_); 89.64 (C-6); 103.64 (C-3a); 111.83 (CN); C_arom_ 120.38 (C-2′ and C-6′), 126.65 (C-4′), 138.42 (C-3′ and C-5′), 140.12 (C-1′),143.42 (C-10a),141.69 (C-3),148.47 (C-5),160.28 (C-9), 161.92 (C-4a); 162.00 (C-7). C_16_H_11_N_7_, 301.1051; HRMS Calcd. for C_16_H_11_N_7_: 301.1076, found: 301.1087.
- *6-Cyano-7-imino-N* ^*1*^ *-phenyl-1,7-dihydropyrazolo[3′,4′:4,5]pyrimido[1,6-a]pyrimidine* ***5d*** Yield 77 %; mp 248 °C; IR (cm^−1^); *ν*_NH_ 3189; *ν*_C≡N_ 2250; *ν*_C=N_ 1532, 1559, 1562; RMN ^1^H (*δ* ppm, DMSO): 7.33 (1H, t, *J* = 7.3 Hz, ArH_4_), 7.55 (2H, t, *J* = 7.3 Hz, ArH_3_ and ArH_5_), 8.03 (1H, s, H_5_), 8.21 (2H, d, *J* = 7.3 Hz, ArH_2_ and ArH_6_), 8.31 (1H, s, H_9_), 8.36 (1H, s, H_3_), 8.37 (1H, s, NH); RMN^13^C (*δ* ppm, DMSO): 89.87 (C-6); 101.37 (C-3a); 120.45 (CN); C_arom_ 126.00 (C-2′ and C-6′), 129.10 (C-4′), 13015 (C-3′ and C-5′), 134.04 (C-1′); 138.94 (C-10a); 139.11 (C-3); 142.14 (C-5);153.19 (C-9); 156.68 (C-4a); 158.26 (C-7); HRMS Calcd. for C_15_H_9_N_7_: 287.0976, found: 287.0919.
- *6-Cyano-7-imino-5-ethyl-N* ^*1*^ *-phenyl-1,7-dihydropyrazolo[3′,4′:4,5]pyrimido[1,6-a]pyrimidine* ***5e*** Yield 70 %; mp 168 °C; IR (cm^−1^); *ν*_NH_ 3332; *ν*_C≡N_ 2218; *ν*_C=N_ 1568, 1589, 1620; RMN ^1^H (*δ* ppm, DMSO): 1.23 (3H, t, CH_3_); 2.30 (2H, q, CH_2_); 7.30 (1H, t, *J* = 7.3 Hz, ArH_4_); 7.52 (2H, t, *J* = 7.3 Hz, ArH_3_ and ArH_5_); 8.04 (2H, d, *J* = 7.3 Hz, ArH_2_ and ArH_6_); 8.18 (1H, s, H_5_); 8.52 (1H, s, H_9_); 11.16 (1H, s, NH); RMN^13^C (*δ* ppm, DMSO): 9.01 (CH_3_): 29.31 (CH_2_); 92.54 (C-6); 106.31 (C-3a); 114.07 (CN); C_arom_ 121.28 (C-2′ and C-6′), 124.73 (C-4′), 126.56 (C-3′ and C-5′), 141.13 (C-1′),145.82 (C-10a),152.63 (C-3),155.28 (C-9),161.23 (C-4a), 162.07 (C-7); 165.49 (C-5); HRMS Calcd. for C_17_H_13_N_7_: 315.1232, found: 315.1352.
- *Ethyl-3,5-dimethyl-7-imino-N* ^*1*^ *-phenyl-1,7-dihydropyrazolo[3′,4′:4,5]pyrimido[1,6-a]pyrimidine-6-carboxylate* ***5f*** Yield 71 %; mp 170 °C; IR (cm^−1^); *ν*_NH_ 3081; *ν*_CO_ 1747; *ν*_C=N_ 1510, 1565, 1590; RMN ^1^H (*δ* ppm, DMSO) 1.21 (3H, t, *J* = 7.2 Hz, CH_3_); 1.91 (3H, s, CH_3_); 2.62 (3H, s, CH_3_); 4.15 (2H, q, *J* = 7.2 Hz, CH_2_); 7.28 (1H, t, *J* = 7.3 Hz, ArH_4_); 7.51 (2H, t, *J* = 7.3 Hz, ArH_3_ and ArH_5_); 8.17 (2H, d, *J* = 7.3 Hz, ArH_2_ and ArH_6_); 8.26 (1H, s, H_9_); 11.97 (1H, s, NH). RMN^13^C (*δ* ppm, DMSO) 13.01 (CH_3_); 14.00 (CH_3_); 24.45 (CH_3_); 66.03 (CH_2_); 105.28 (C-6); 115.10 (C-3a); 121.07 (C-2′ and C-6′), 125.50 (C-4′), 129.12 (C-3′ and C-5′), 138.88 (C-1′),142.79 (C-10a),146.88 (C-3), 148.30 (C-5), 154.14 (C-9), 156.21 (C-4a), 156.48 (C-7), 164.27 (CO); HRMS Calcd. for C_19_H_18_N_6_O_2_: 362.1491, found: 362.1478.
- *Ethyl-5-ethyl-7-imino-3-methyl-N* ^*1*^ *-phenyl-1,7-dihydropyrazolo[3′,4′:4,5]pyrimido[1,6-a]pyrimidine-6-carboxylate* ***5g*** Yield 69 %; mp 181 °C; IR (cm^−1^); *ν*_NH_ 3081; *ν*_CO_ 1706; *ν*_C=N_ 1434, 1493, 1589; RMN ^1^H (*δ* ppm, DMSO) 1.06 (3H, t, *J* = 7.1 Hz, CH_3_); 1.34 (3H, t, *J* = 7.0 Hz, CH_3_); 1.97 (2H, q, *J* = 7.1 Hz, CH_2_); 2.63 (3H, s, CH_3_); 4.03 (2H, q, *J* = 7.0 Hz, CH_2_); 7.49 (1H, t, *J* = 7.3 Hz, ArH_4_); 7.63 (2H, t, *J* = 7.3 Hz, ArH_3_ and ArH_5_); 8.03 (2H, d, *J* = 7.3 Hz, ArH_2_ and ArH_6_); 9.57 (1H, s, H_9_); 11.96 (1H, s, NH). RMN^13^C (*δ*ppm, DMSO) 11.26 (CH_3_); 14.03 (CH_3_); 14.07 (CH_3_); 30.19 (CH_2_); 67.92 (CH_2_); 105.58 (C-6); 114.96 (C-3a); 120.64 (C-2′ and C-6′), 125.99 (C-4′), 129.69 (C-3′ and C-5′), 139.45 (C-1′),143.25 (C-10a),154.76 (C-3), 156.97 (C-5), 159.15 (C-9), 162.04 (C-4a), 162.50 (C-7), 164.09 (CO); HRMS Calcd. for C_20_H_20_N_6_O_2_: 376.1648, found 376.1621.
- *Ethyl-7-imino-N* ^*1*^ *-phenyl-1,7-dihydropyrazolo[3′,4′:4,5]pyrimido[1,6-a]pyrimidine carboxylate* ***5h*** Yield 89 %; mp 184 °C; IR (cm^−1^); *ν*_NH_ 3227; *ν*_CO_ 1710; *ν*_C=N_ 1539, 1552, 1574.17; RMN ^1^H (*δ* ppm, DMSO) 1.29 (3H, t, *J* = 7.0 Hz, CH_3_); 4.24 (2H, q, *J* = 7.0 Hz, CH_2_); 7.37 (1H, t, *J* = 7.3 Hz, ArH_4_); 7.55 (2H, t, *J* = 7.3 Hz, ArH_3_ and ArH_5_); 8.14 (2H, d, *J* = 7.3 Hz, ArH_2_ and ArH_6_); 8.75 (1H, s, H_5_); 8.83 (1H, s, H_9_); 9.18 (1H, s, H_3_); 12.11 (1H, s, NH). RMN^13^C (*δ* ppm, DMSO) 14.11 (CH_3_); 61.36 (CH_2_); 103.83 (C-6); 114.46 (C-3a); 120.62 (C-2′ and C-6′), 126.73 (C-4′), 129.20 (C-3′ and C-5′), 134.35 (C-1′),138.10 (C-10a),148.14 (C-3), 151.37 (C-5), 153.53 (C-9), 154.00 (C-4a), 155.18 (C-7), 163.36 (CO). 120.62-126.73-129.20-134.35, C_17_H_14_N_6_O_2_, 334.1171; HRMS Calcd. for: C_17_H_14_N_6_O_2_: 334.1178, found: 334.1171.
- *Ethyl-5-methyl-7-imino-N* ^*1*^ *-phenyl-1,7-dihydropyrazolo[3′,4′:4,5]pyrimido[1,6-a] pyrimidine-6-carboxylate* ***5i*** Yield 78 %; mp 166 °C; IR (cm^−1^); *ν*_NH_ 3059; *ν*_CO_ 1718; *ν*_C=N_ 1579, 1591, 1612; RMN ^1^H (*δ* ppm, DMSO) 1.34 (3H, t, *J* = 7.0 Hz, CH_3_); 1.92 (3H, s, *J* = 7.1 Hz, CH_3_); 4.02 (2H, q, *J* = 7.0 Hz, CH_2_); 7.30 (1H, t, *J* = 7.3 Hz, ArH_4_); 7.61 (2H, t, *J* = 7.3 Hz, ArH_3_ and ArH_5_); 8.10 (2H, d, *J* = 7.3 Hz, ArH_2_ and ArH_6_); 9.29 (1H, s, H_3_); 9.49 (1H, s, H_9_); 11.95 (1H, s, NH). RMN^13^C (*δ* ppm, DMSO); 15.06 (CH_3_); 23.14 (CH_3_); 69.54 (CH_2_); 102.85 (C-3a); 117.05 (C-6); 121.637 (C-2′ and C-6′), 126.41 (C-4′), 128.65 (C-3′ and C-5′), 139.24 (C-1′),143.92 (C-10a),144.17 (C-3), 159.62 (C-5), 161.45 (C-9), 167.12 (C-4a), 167.83 (C-7), 168.28 (CO); HRMS Calcd. for C_18_H_16_N_6_O_2_: 348,1335, found 348,1274.

### Pharmacology

Carrageenan (BDH Chemicals Ltd., Poole, England), cimetidine and acetylsalicylic–lysine were purchased from pharmacie Centrale of Tunisia.

#### Animals

Adult Male Wistar rats weighing 150–170 g were obtained from Pasteur Institute (Tunis, Tunisia). They were housed in polypropylene cages and left for 2 days for acclimatization to animal room maintained under controlled conditions: a 12 h light–dark cycle (at 22 ± 2 °C) on standard pellet diet and water ad libitum. Rats were fasted overnight with free access to water before the experiments. Housing conditions and in vivo experiments were approved, according to the guidelines established by the European Union on Animal Care (Communautés Économiques Européennes Council [86/609]).

#### Carrageenan-induced rat paw oedema

The anti-inflammatory activity of compounds (**5a**, **b**, **f**, **g**) on carrageenan-induced rat paw oedema was determined according to Winter *et al.* (1962). The animals were divided into three groups of six rats each. The control group received intraperitoneally 2.5 ml/kg of vehicle solution (Tween 80/absolute ethanol/saline solution (0.9 %) in the ratio 1:1:18). The reference group received acetylsalicylic–lysine (300 mg/kg i.p.), and the test groups received compounds **5a**, **b**, **f**, **g** (50 and 100 mg/kg, i.p.). After 30 min, 0.05 ml of 1 % carrageenan suspension was injected into the left hind paw. The paw volume up to the tibiotarsal articulation was measured using a plethysmometer (model 7150, UgoBasile, Italy) at 0 h (*V*_0_) (before carrageenan injection) and 1, 3 and 5 h later (*V*_T_) (after carrageenan injection). Paw swelling was determined for each rat and the difference between *V*_T_ and *V*_0_ was taken as the oedma value. The percent inhibition was calculated according to the following formula:

$$ {\text{\% Inhibition:}}\,\left[ {\left( {V_{\text{T}} - V_{ 0} } \right)_{\text{control}}\, - \,\left( {V_{T} - \, V_{ 0} } \right)_{\text{treated}} } \right] \, \times 1 0 0/\left( {V_{\text{T}} - V_{ 0} } \right)_{\text{control}} $$

#### Gastroprotective activity

The gastroprotective activity of pyrazolopyrimidopyrimidines **5a**, **b**, **f**, **g** was studied in 150 mM HCl/EtOH-induced gastric ulcer (Hara and Okabe, 1985). Rats were fasted for 24 h prior receiving any treatment and were divided into six groups of six animals each. Group I was kept as control group and received the vehicle (Tween 80/Absolute ethanol/Saline solution (0.9 %): 1/1/18). Group II and III received compound **5a** (50, 100 mg/kg, i.p.), respectively, and Group IV and V received compound **5b** (50, 100 mg/kg, i.p.), respectively. Group VI and VII received compound **5f** (50, 100 mg/kg, i.p.), respectively, and group VIII and IX received compound **5g** (50, 100 mg/kg, i.p.), respectively. Group X received cimetidine (100 mg/kg, i.p.) as reference drug. After 30 min, all groups were orally treated with 1 ml/100 g of 150 mM HCl/EtOH (40:60, v/v) solution for gastric ulcer induction. Animals were sacrificed 1 h after the administration of ulcerogenic agent; their stomachs were excised and opened along the great curvature, washed and stretched on cork plates. The surface was examined for the presence of lesions and the extent of the lesions was measured. The summative length of the lesions along the stomach was recorded (mm) as lesion index.

### Statistics

Results are expressed as the mean ± SEM of six animals per group. The data were analysed using Student’s *t* test, *p < 0.05, **p < 0.01 and ***p < 0.001 was considered significant.

## Results and discussion

### Chemistry

**Scheme 1:**
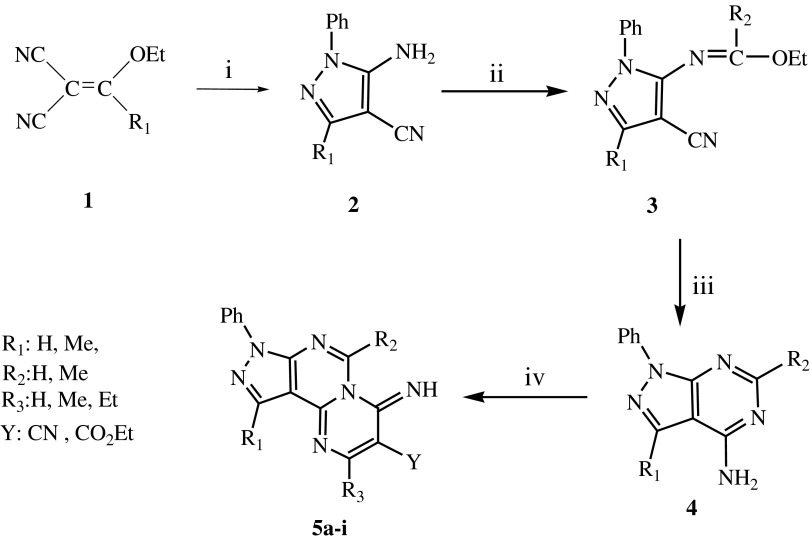
Synthetic procedure of compounds **5a**–**i**. Reagents: *i* H_2_N–NHPh, CH_3_CO_2_H, CH_3_CO_2_H; *ii* R_2_C(OEt)_3_, CH_3_CO_2_H; *iii* NH_3_; *iv*

The synthetic routes to target compounds **5a**–**i** are outlined in Scheme 1. The 5-amino-4-cyano-*N* ^1^-phenylpyrazole **2**, used as a starting material, was prepared in two steps following a similar method reported by Petrie *et al.* (1985), Anderson *et al.,* (1990), Aggarwal *et al.,* (2011). The first step involves acid-catalysed condensation of orthoester with malonate to form ethoxymethylene malononitrile **1**. This later reacts then with substituted hydrazine to give the aminocyanopyrazole **2**. Treatment of **2** with orthoester in the presence of catalytic amount of acid furnished the corresponding cyano-pyrazoloimidates **3** which subsequently were transformed to the corresponding amino pyrazolopyrimidines **4** (Booth *et al.*, 1999; Gupta *et al.*, 2008; Oliveira-Campos *et al.*, 2007; Bakavoli *et al.*, 2010) upon treatment with ammoniac. Reaction of compound **4** with ketene ethoxymethylene compounds **1** in ethanol in presence of catalytic amount of acid furnished the desired 6-cyano-1,7-dihydropyrazolo[3′,4′:4,5]pyrimido[1,6-*a*]pyrimidine **5a**–**e** in 70 % yield as a yellow solid. The same procedure gave a crystalline ethyl-1,7-dihydro pyrazolo [3′,4′:4,5]pyrimido [1,6-*a*]pyrimidine-6-carboxylate **5f**–**i** from ethyl-2-cyano-3-ethoxyalkyl-2-enoate in 80 % yield. Scheme 1 shows the synthetic strategy to obtain the target compounds by the four-steps method, yielding the compounds with structure **5a**–**i** listed in Table 1.

**Table 1:** Synthesis of 7-imino-*N* ^1^-phenyl-1,7-dihydro pyrazolo[3′,4′:4,5]pyrimido[1,6-*a*]pyrimidine **5a**–**i**

Compounds	R_1_	R_2_	R_3_	Y	Yields (%)	Reaction time (h)
**5a**	CH_3_	H	H	CN	68	24
**5b**	CH_3_	H	CH_3_	CN	54	71
**5c**	H	CH_3_	H	CN	71	24
**5d**	H	H	H	CN	77	5
**5e**	H	H	C_2_H_5_	CN	70	48
**5f**	CH_3_	H	CH_3_	CO_2_Et	71	75
**5g**	CH_3_	H	C_2_H_5_	CO_2_Et	69	84
**5h**	H	H	H	CO_2_Et	89	7
**5i**	H	H	CH_3_	CO_2_Et	78	24

It is interesting to note that time reaction and yield of products are directly related to the nature of substituent (R_3_ and Y). The yields of compounds **5h** and **5d** are 89 and 77 %, respectively. Hydrogen substituent R^3^ gave superior yields in short time. In all cases, reaction leads to pyrazolo pyrimido pyrimidine only when R^1^ or R^2^ is a hydrogen atom. However, steric effect decreased yields of the reaction, as in the case of **5g**, and may even prevent the progress of the reaction when R^2^ and R^3^ are methyl groups. Analysis of the NMR and IR spectra indicated that compounds **5f**–**i** has ester functional group in their structures so ethoxymethylene cyanoacetate reacts with pyrazolopyrimidine and in both cases Y is CN or CO_2_Et, nitrogen attacked on the nitrile function as the first attack.

### Biological activity

#### Anti-inflammatory and gastroprotective activities of compounds **5a**, **b**, **f**, **g**

**Fig. 1:**
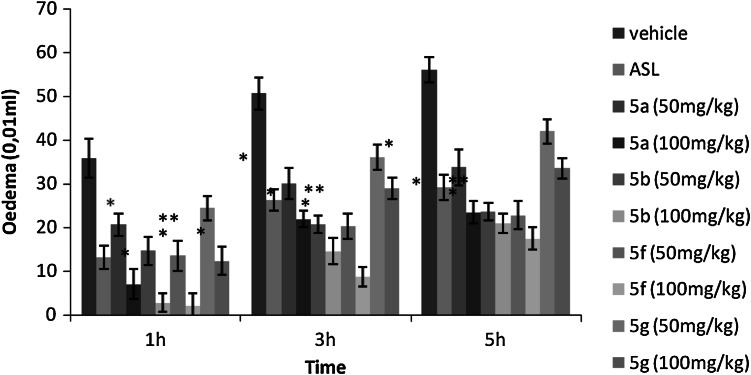
Anti-inflammatory effect of the intraperitoneal administration of** 5a**,** b**,** f**,** g** and of the reference drug (acetylsalicylic–lysine: ASL) in carrageenan-induced rat paw oedema. The values represent the means difference of volume of paw ± SEM (*n* = 6). *p < 0.01 and **p < 0.001 significantly different from the control group

**Fig. 2:**
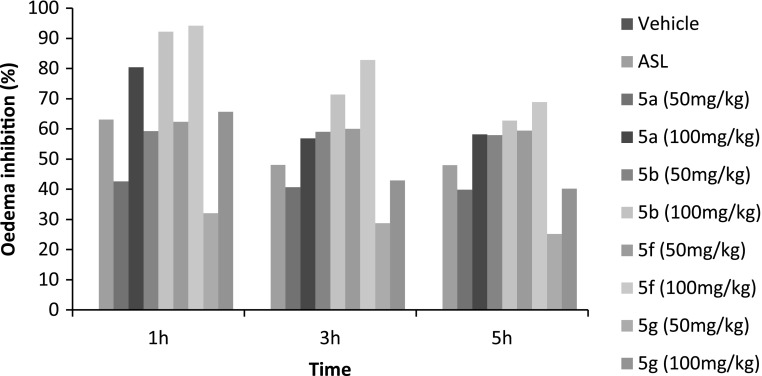
Percentage inhibition of the oedema after the intraperitoneal administration of** 5a**,** b**,** f**,** g** and the reference drug (acetylsalicylic–lysine: ASL) in carrageenan-induced rat paw oedema

The pyrazolopyrimidine derivatives are a well-known class of NSAIDs with several products in market (Russo *et al.*, 1992; El-Kateb *et al.*, 2012) (Figs. 1, 2).

The structure–activity relationships (SAR) for these compounds have been extensively explored for optimization of anti-inflammatory activity last three decades, since this class was introduced (Lombardino and Wiseman, 1972; Farré *et al.*, 1986; Berq *et al.*, 1999; Lee *et al.*, 1999). In continual efforts to find potentially safer and more efficacious parent agents through further exploration of SAR of this class, we decided to study the pharmacological profiles of compounds **5a**, **b**, **f**, **g** belonging to pyrazolopyrimidopyrimidine family. We examined the effect of modification of the electronic nature of substituents on various portions of type NSAIDs. For this objective the hydrogen atom (position 5) is replaced by methyl or ethyl group, even and for more important anti-inflammatory activity, the cyano function is replaced by ester function.

Table 2 reveals the results of the intraperitoneal administration of the compounds **5a**, **b**, **f**, **g** in carrageenan-induced rat paw oedema. The compounds **5a**, **b**, **f**, **g** tested at 50 and 100 mg/kg, i.p. produced a significant reduction of the oedema throughout the entire period of observation in a dose-dependent manner. The highest reduction of the oedema was at 3 h after carrageen injection with a percent inhibition ranged, from 40.64 to 56.81 % for compound **5a**, from 58.98 to 71.36 % for compound **5b**, from 60.02 to 82.83 % for compound **5f** and from 28.75 to 42.87 % for compound **5g**, whereas the reference drug (acetylsalicylic–lysine, 300 mg/kg, i.p.) produced 48.03 % reduction in paw volume. The influence of the substituent R_2_ on activity is remarkable. Compound **5a** is less potent than the 5-methyl derivatives **5b**, so a methyl group linked to the pyrimidine cycle increases the activity compared to the case of a hydrogen atom. At the same dose (100 mg/kg), compound **5b** produced 71.36 % inhibition of oedema against 56.81 % for **5a**. In addition, the compound **5f** is more potent than the ethyl derivatives **5g**, so an ethyl group linked to the pyrimidine cycle decreases the activity compared to the methyl group.

**Table 2:** Anti-inflammatory effect of the intraperitoneal administration of **5a**, **b**, **f**, **g** and of the reference drug (acetylsalicylic–lysine: ASL) in carrageenan-induced rat paw oedema

Sample	Dose (mg/kg)	Oedema (10^−2^ ml) (mean ± SEM)			Oedema inhibition (%)		
		1 h	3 h	5 h	1 h	3 h	5 h
Vehicle (2,5 ml/kg)	–	35.87 ± 4.48	50.66 ± 3.68	56.04 ± 2.91	–	–	–
Acetylsalicylic–lysine (reference drug)	300	13.23 ± 2.69**	26.32 ± 2.44**	29.15 ± 2.87**	63.10	48.03	47.98
**5a**	50	20.59 ± 2.51*	30.07 ± 3.51*	33.73 ± 4.16*	42.59	40.64	39.8
	100	7.01 ± 3.41**	21.88 ± 1.89**	23.45 ± 2.5**	80.44	56.81	58.15
**5b**	50	14.62 ± 3.21*	20.78 ± 2*	23.56 ± 2*	59.25	58.98	57.95
	100	2.81 ± 2.06***	14.51 ± 2.98***	20.86 ± 2.21***	92.17	71.36	62.76
**5f**	50	13.51 ± 3.4**	20.25 ± 2.8**	22.74 ± 3.2**	62.31	60.02	59.42
	100	2.07 ± 2.8***	8.69 ± 2.3***	17.45 ± 2.5***	94.22	82.83	68.85
**5g**	50	24.37 ± 2.7*	36.09 ± 2.9*	41.95 ± 2.8	32.04	28.75	25.13
	100	12.31 ± 3.2**	28.94 ± 2.4*	33.52 ± 2.3	65.66	42.87	40.18

The values represent the mean difference of volume of paw ± SEM (*n* = 6)

* p < 0.05, ** p < 0.01, *** p < 0.001 significantly different from control group

**Fig. 3:**
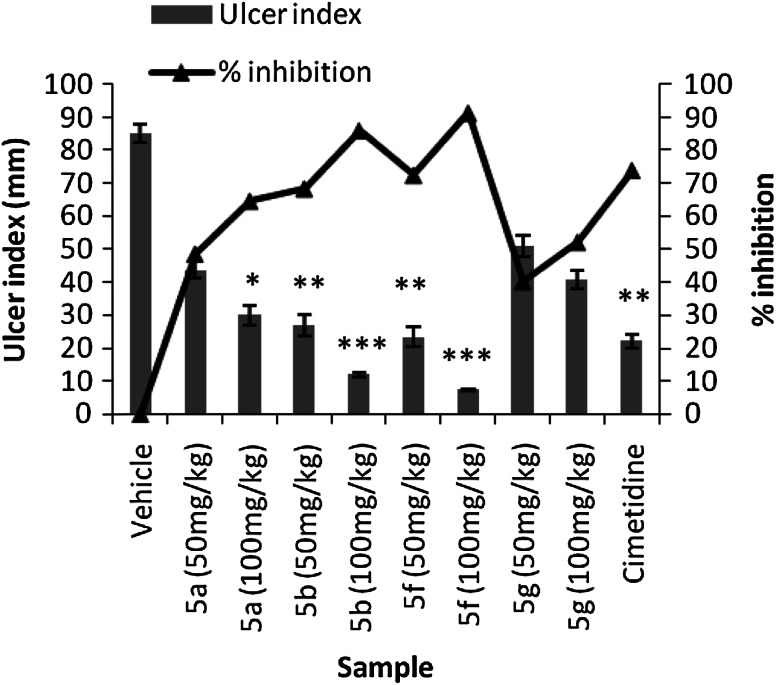
Effect of compounds** 5a**,** b**,** f**,** g** and the reference drug (cimetidine) on gastric ulcer induced by HCl/ethanol in rats. Data expressed as mean ± SEM (*n* = 6). *p < 0.05, **p < 0.01, ***p < 0.001 significantly different from control group

On the other hand, mucosal erosion and ulceration are produced by most NSAIDs with varying degrees. Inhibition of synthesis of gastroprotective prostaglandins (PGE_2_) is clearly involved (Nezamis *et al.*, 1967) and due to the inhibition of the constitutive isoform COX-1 (Main and Whittle, 1973; Cryer and Feldman, 1992). Thus, deficiency of PGs reduces the mucosal secretions along with hydrogen carbonate that ultimately aggravates the lethal effects of acid on the stomach lining leading to mucosal damage (Fig. 3).

The results of gastroprotective activity of compounds **5a**, **b**, **f**, **g** on gastric ulcer induced by HCI/ethanol solution are shown in Table 3. Oral administration of the ulcerogenic agent to the control group clearly showed a mucosal damage characterized by multiple haemorrhage red bands of different sizes along the long axis of the glandular stomach as described in other studies (Shay *et al.*, 1945; Yassir *et al.*, 1999). When we compared the gastroprotective activity of compounds **5a**, **b**, **f**, **g** we observed that pyrazolopyrimidopyrimidine **5b** (100 mg/kg) demonstrated the higher significant inhibition of gastric lesion (91, 42 %).

**Table 3:** Effect of compounds **5a**,** b**,** f**,** g** and the reference drug (cimetidine) on gastric ulcer induced by HCl/ethanol in rats

Treatment	Dose (mg/kg)	Ulcer index (mm)	Inhibition (%)
Vehicle (2.5 ml/kg) (control)	–	85 ± 2.82	–
Compounds			
**5a**	50	43.66 ± 2.58	48.63
	100	30 ± 3.03*	64.7
**5b**	50	26.83 ± 3.43**	68.43
	100	11.83 ± 0.75***	86.08
**5f**	50	23.34 ± 2.9**	72.53
	100	7.29 ± 0.3***	91.42
**5g**	50	50.81 ± 3.2	40.22
	100	40.65 ± 2.8	52.17
Cimétidine (reference drug)	100	22.07 ± 2.12**	74.03

Data expressed as mean ± SEM (*n* = 6)

* p < 0.05, ** p < 0.01, *** p < 0.001 significantly different from control group

In conclusion, we have synthesized a new series of 1,7-dihydropyrazolo [3′,4′:4,5]pyrimido[1,6-*a*]pyrimidine **5a**–**i** derivatives. The yield of the reaction seems to be significantly influenced by the nature of substituent. The highest yield is obtained for more hydrogen atom substituent. However, test (or experimental) compounds **5a**, **b**, **f** showed that the methyl group increases the anti-inflammatory activity, contrary to ethyl group which decreases this activity. The same interpretation is found with gastroprotective effect. Indeed, our results on the gastroprotective effects of compounds **5a**, **b**, **f** compared with cimetidine indicate that replacement of hydrogen by methyl reduces the gastrointestinal adverse effects.
